# Beyond insulin-like growth factor-1 (IGF-1) normalization: portal insulin and hepatic growth hormone (GH) sensitivity across metabolic disease

**DOI:** 10.20945/2359-4292-2026-0115

**Published:** 2026-09-16

**Authors:** Aart J. van der Lely, Sebastian J. Neggers, John J. Kopchick, Cesar L. Boguszewski

**Affiliations:** 1 Pituitary Center Rotterdam, Department of Medicine, Erasmus University Medical Center, Rotterdam, The Netherlands; 2 Edison Biotechnology Institute and Heritage College of Osteopathic Medicine, Ohio University, Athens, USA; 3 Serviço de Endocrinologia e Metabologia do Paraná, Divisão de Endocrinologia, Departamento de Medicina Interna, Universidade Federal do Paraná, Curitiba, PR, Brasil

**Keywords:** Growth hormone, insulin-like growth factor-1, insulin, portal vein, acromegaly, diabetes mellitus, liver cirrhosis, GLP-1 receptor agonists

## Abstract

Clinical management of growth hormone (GH) deficiency, acromegaly, and diabetes mellitus is dominated by single-analyte targets: serum insulin-like growth factor-1 (IGF-1) in the GH disorders and glycated hemoglobin in diabetes. This reductive framework obscures a central regulatory fact: GH does not autonomously dictate hepatic IGF-1 output. Whether GH is translated into IGF-1 by the hepatocyte is gated by the concentration of insulin reaching the liver through the portal vein. We propose liver sensitivity to GH (LSG), operationally defined as portal-insulin-determined hepatic GH receptor (GHR) availability, as a unifying variable that places type 1 and type 2 diabetes, obesity, liver failure, and acromegaly on a single continuum rather than treating them as separate phenomena. We develop the molecular basis of insulin-dependent GHR regulation, distinguish the divergent roles of portal versus systemic insulin, and argue that this distinction explains why subcutaneous insulin therapy in type 1 diabetes corrects glycemia while leaving the hepatic GH-resistant state intact. We then re-examine the weight-gain liability of insulinotropic antidiabetic agents, the discordant GH/IGF-1 signatures produced by GLP-1 receptor agonists, the prognostic and potentially therapeutic role of IGF-1 in cirrhosis, and the metabolic limitations of current acromegaly treatment targets when viewed through the LSG framework. The aim is to give the treating physician a mechanistic, rather than purely biochemical, account of how metabolism behaves in these states.

## INTRODUCTION

Three of very familiar endocrine conditions, growth hormone deficiency (GHD), acromegaly, and diabetes mellitus, are managed as though each were a single-dial problem. Growth hormone (GH) deficiency is dosed to normalize serum IGF-1; acromegaly is treated to lower GH or block GH action until insulin-like growth factor-1 (IGF-1) falls within the age- and sex-adjusted reference range; type 1 diabetes is dosed with insulin until glycated hemoglobin normalizes (^1^-^4^). The clinical appeal is obvious: each disease acquires a clean efficacy metric, and competing therapies can be ranked by the proportion of patients reaching target.

The cost of this convenience is conceptual. GH, IGF-1, and insulin do not act in parallel; they continuously rewrite one another’s production and action (^5^-^7^). Treating IGF-1 as a faithful readout of GH status, or glycated hemoglobin as a complete description of metabolic control, ignores the variable that couples the two systems together. That variable is the amount of insulin reaching the liver through the portal circulation. In this review we make this coupling explicit and have named it liver sensitivity to GH (LSG), and use it to connect disorders that are conventionally taught in isolation. We focus on type 1 and type 2 diabetes, obesity, liver failure, and acromegaly as the settings in which a clinician who reasons only from IGF-1 or glucose levels will be misled.

## DEFINING LIVER SENSITIVITY TO GROWTH HORMONE

LSG is best understood through an electrical analogy. Picture each hepatocyte as a light bulb whose filament is the JAK2/STAT5 cascade that drives IGF-1 transcription. GH is the current flowing into the bulb. Portal insulin is the dimmer switch on the wall. The same current produces a bright or a dim light depending entirely on where the dimmer is set. Crank the dimmer up (high portal insulin) and a small current yields a bright output; turn it down (low portal insulin) and even a large current barely registers.

This is not metaphorical handwaving. Insulin controls the expression of human hepatic GH receptors (GHRs) (^8^). In the HuH7 hepatoma model, Leung and cols. showed that insulin upregulates total and intracellular GHRs concentration-dependently and increases cell-surface GHRs expression in a biphasic fashion, with a peak at approximately 10 nmol/L, modulating GH-induced JAK2 phosphorylation in parallel with surface receptor abundance (^8^). Surface GHR availability is the net result of divergent insulin actions on biosynthesis and translocation, mediated respectively by the MAPK and PI3K pathways (^8^). The dimmer, in other words, dictates the number of GHRs that reach the hepatocyte surface to catch the GH signal.

Defining LSG as portal-insulin-determined hepatic GHR availability converts four disease vignettes into four positions on one axis. Low-LSG states (type 1 diabetes, end-stage liver disease, prolonged fasting) feature scarce portal insulin, relatively low GHR expression by the hepatocytes, thus GH-resistant livers, low IGF-1, and a compensatory rise in GH. High-LSG states (type 2 diabetes, obesity, acromegaly) feature abundant portal insulin, relatively high GHR expression by the hepatocytes, thus GH-hypersensitive livers, and IGF-1 output that outruns what the prevailing GH concentration would otherwise produce. The clinical signatures that follow are not a list of unrelated facts but predictable consequences of the dimmer setting.

## MOLECULAR GATEKEEPING: HOW INSULIN SETS THE DIMMER

Insulin and IGF-1 both suppress GH at the somatotroph, but by separate mechanisms. Using Cre/loxP knockdown of the insulin receptor and the IGF-1 receptor in primary mouse pituitary cultures, Gahete and colleagues showed that insulin-mediated suppression of GH is independent of the IGF-1 receptor, and pharmacological dissection in mouse and baboon cultures confirmed that insulin and IGF-1 recruit different intracellular routes to inhibit pituitary GH secretion (^5^). The two signals converge on the same output but travel different roads.

At the hepatocyte, the decisive event is GHR surface translocation. Insulin’s biphasic effect on surface GHRs, with divergent MAPK-driven biosynthesis and PI3K-driven translocation, means that the hepatic GH sensitivity is set post-transcriptionally and dynamically, on a timescale fast enough to respond to feeding and fasting (^8^). Superimposed on this is SIRT1, which negatively regulates GH-induced IGF-1 production by inhibiting STAT5 activation, providing a nutrient-sensing brake that produces GH resistance during fasting, starvation, and malnutrition (^6^).

A second, faster amplifier operates through IGF-binding protein-1 (IGFBP-1). Insulin acutely suppresses hepatic IGFBP-1 secretion; because IGFBP-1 restrains IGF-1 action, high portal insulin raises not only total IGF-1 generation but also the free, bioactive fraction (^9^,^10^) The dimmer therefore has two ganged controls: GHR availability (how much IGF-1 is made) and IGFBP-1 production (how much of it is active).

The buffering architecture of the circulation matters for interpretation. Six high-affinity IGFBPs regulate IGF-1; in serum, IGFBP-3 and IGFBP-5 are held in a ternary complex with the acid-labile subunit, slowing clearance such that total circulating IGF-1 in adults sits near 100 nM, roughly a thousandfold higher than free insulin (^9^). Both ligands can cross-activate each other’s receptors at reduced affinity (^11^). Consequently, when portal insulin is low, the liver must limit IGF-1 production to prevent IGF-1 from inducing hypoglycemia through the insulin receptor. It accomplishes this precisely by reducing hepatic GHR expression (^8^).

## THE PORTAL-SYSTEMIC INSULIN GRADIENT: THE CENTRAL ARGUMENT

The single most clinically consequential point in this review is that portal insulin and systemic insulin are not interchangeable. The hepatocyte is bathed by portal blood; it never directly samples the systemic concentration that subcutaneous injection or peripheral assay reports. A recent update by the same conceptual lineage has formalized this, arguing that intra-portal insulin is the proximate regulator of hepatic GH sensitivity and that discordant GH/IGF-1 profiles across disease states are best read as a “secondary association” driven by portal insulinization rather than by GH secretion itself (^12^).

This distinction dissolves several apparent paradoxes. In type 1 diabetes, subcutaneous insulin restores systemic concentrations but leaves the portal vein relatively insulin-poor; the liver stays GH-resistant even when glucose is controlled. During peritoneal dialysis, insulin delivered in the dialysate or intraperitoneally reaches the portal circulation more directly, partially restoring hepatic GHR expression, which is one reason intraperitoneal dosing requirements differ from subcutaneous (^13^). In acromegaly, the effect of the systemic hyperinsulinemia is more than adequate to counter GH’s peripheral lipolysis, yet the high portal insulin still fails to neutralize hepatic GH drive as the liver behaves as supersensitive for GH, so gluconeogenesis persists (^14^). The recurring therapeutic error, treating a portal-compartment problem with a systemic-compartment intervention, becomes visible only once the gradient is made explicit.

## SYSTEMS-LEVEL FEEDBACK AND MODIFIERS

Closing the loop, IGF-1 feeds back on GH secretion through GHRH-dependent hypothalamic mechanisms, demonstrated by pharmacologic and genetic-mouse interventions (^15^). The net physiological circuit: insulin raises hepatic GHR expression, making the liver GH-sensitive, increasing IGF-1 and thereby suppressing pituitary GH; low portal insulin during fasting reduces GHR expression, lowering IGF-1 and releasing pituitary GH from feedback restraint.

**Figure 1:**
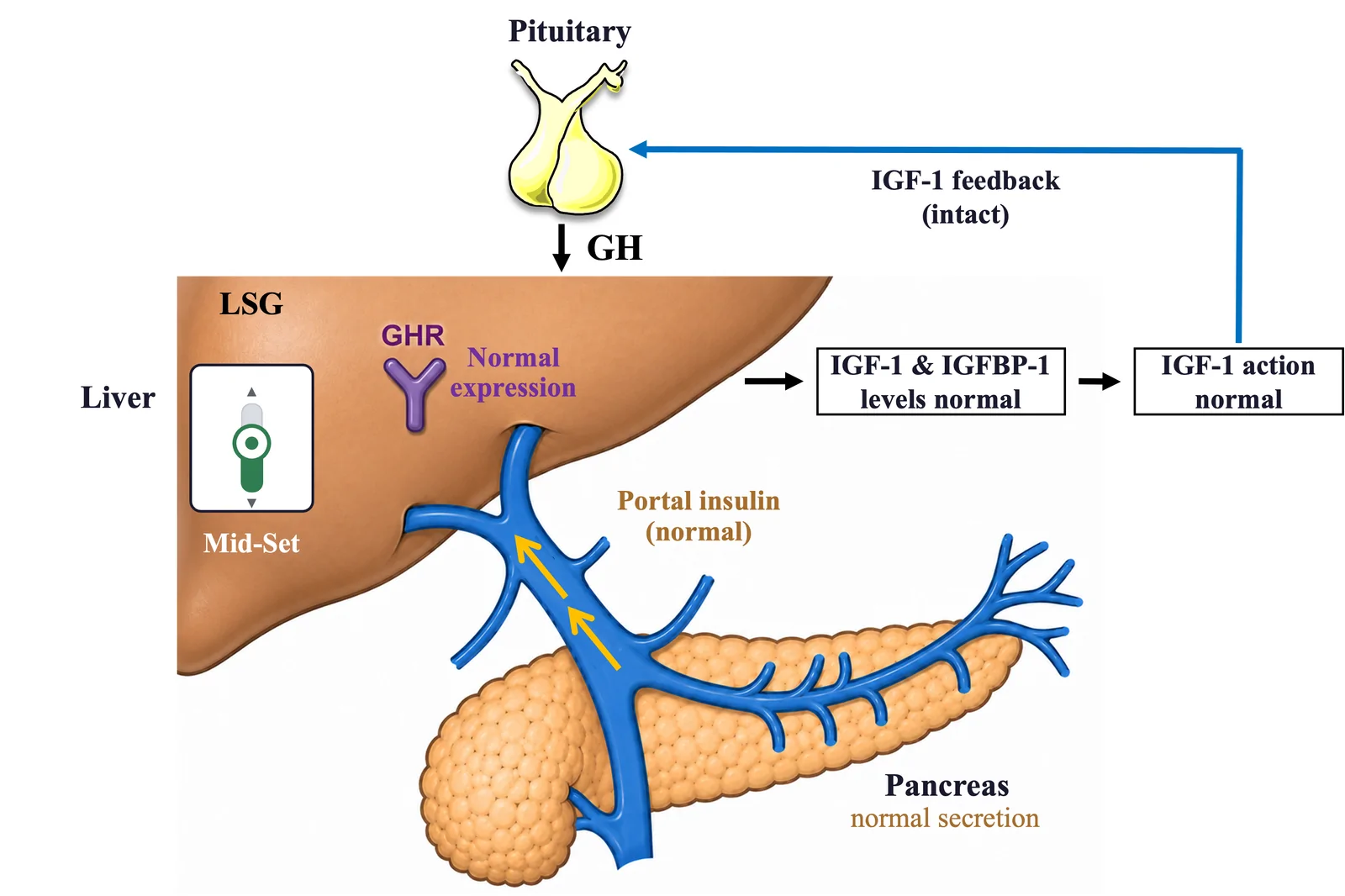
**Normal physiology.** Portal insulin holds hepatic GHR availability (LSG) at a physiological midpoint, so pituitary GH is translated into hepatic IGF-1 in proportionate fashion and circulating IGF-1 is normal. IGF-1 exerts GHRH-dependent negative feedback on the pituitary, restraining GH secretion; the loop is self-correcting and no arm dominates. GH: growth hormone; GHR: GH receptor; IGF-1: insulin-like growth factor-1; IGFBP-1: IGF-binding protein-1; LSG: liver sensitivity to GH (portal-insulin-set hepatic GHR availability). Arrow thickness denotes the magnitude of each flux; the vertical LSG slider denotes hepatic GH-receptor availability; the orange arrows are the portal insulin setting the slider; the blue line denotes the intact IGF-1 negative feedback on the pituitary.

Two modifiers deserve mention because they alter the dimmer’s calibration. Sex steroids and the metabolic partitioning of GH itself. In GHD, women require higher GH replacement doses than men because androgens enhance and estrogens attenuate GH action, an effect that is route-dependent, with oral estrogen blunting liver-mediated GH action by directly inhibiting GHR signaling (^16^). Second, the metabolic partitioning of GH itself: GH is anabolic in most tissues but catabolic in adipose tissue, where it promotes lipolysis, splitting triglycerides into free fatty acids. Chronic GH excess suppresses insulin’s anti-lipolytic action, increasing free fatty acid flux and promoting lipotoxic insulin resistance (^10^). The dimmer analogy still applies, but the bulb is wired to different fixtures in different tissues (**Figure 1**).

## LOW-LSG STATES I: TYPE 1 DIABETES AND THE MISDIRECTED COMPARTMENT

Type 1 diabetes is the cleanest low-LSG state. Because endogenous insulin production fails, portal insulin is low even under subcutaneous therapy, which raises only systemic levels. The classic intraportal-infusion work of Shishko and colleagues in recent-onset type I diabetes patients showed that IGF-1 normalization depends on both glycemic control and the route of insulin delivery: only when insulin was returned to the portal vein did serum IGF-1, IGFBP-1, and GH normalize (^17^).

**Figure 2:**
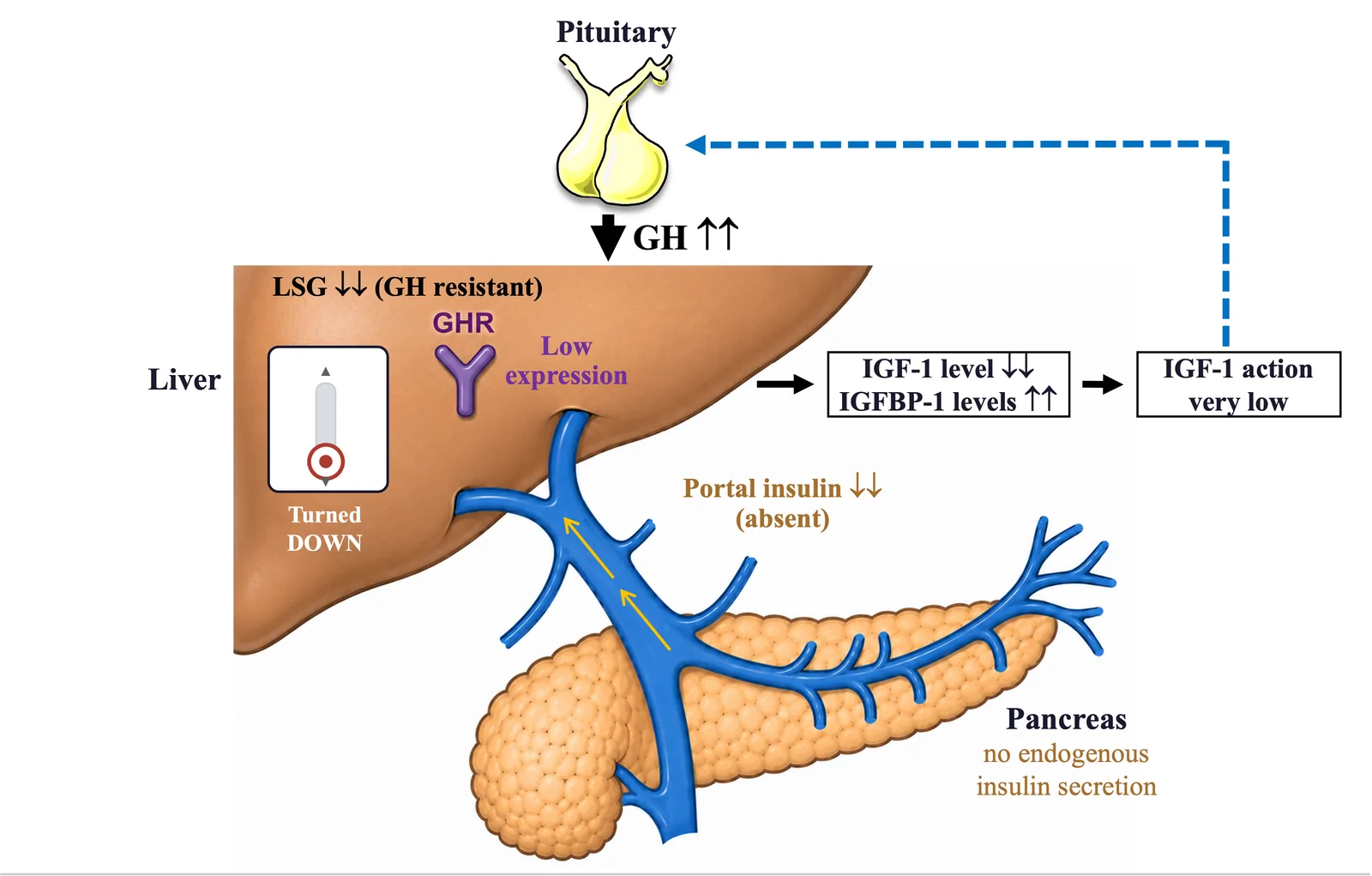
**Type 1 diabetes mellitus.** Absent endogenous insulin leaves the portal vein insulin-poor even when subcutaneous therapy normalizes systemic insulin, so the LSG slider is turned down and the liver is GH-resistant. Hepatic IGF-1 output collapses; loss of IGF-1 negative feedback releases pituitary GH into the acromegaly range. Correcting glycemia does not relieve hepatic GH resistance—only portal (e.g., intraperitoneal) insulin delivery restores the slider. GH: growth hormone; GHR: GH receptor; IGF-1: insulin-like growth factor-1; IGFBP-1: IGF-binding protein-1; LSG: liver sensitivity to GH (portal-insulin-set hepatic GHR availability). Arrow thickness denotes the magnitude of each flux; the vertical LSG slider denotes hepatic GH-receptor availability; the orange arrows are the portal insulin setting the slider; the dotted blue arrow denotes the impaired IGF-1 negative feedback on the pituitary.

The functional consequence is striking. The type 1 diabetic liver behaves as if fasting even while the patient eats: it encounters portal-borne nutrients but no portal insulin, and the absence of insulin overrides the presence of nutrients as a signal, so the hepatocyte runs gluconeogenesis and lipolysis postprandially. Systemic insulin lowers glucose but does not relieve hepatic GH resistance; only portal delivery addresses the underlying derangement (^17^,^18^). In end-stage renal disease requiring peritoneal dialysis, providing insulin via dialysate restores hepatic GHR expression, permits GH-driven IGF-1 recovery, restores feedback on GH, and thereby improves whole-body insulin sensitivity, lowering the insulin dose needed for control (^13^) (**Figure 2**).

Wurzburger and cols. analyzed the IGF-I concentrations before and after 7 days treatment with recombinant human GH in diabetes subjects (^19^). In case of absence of C-peptide (CpN), patients were considered to have no endogenous pancreatic beta-cell activity. Patients who were C-peptide positive (CpP) still had endogenous insulin secretion. The net increase in IGF-I concentrations after rhGH treatment was significantly lower in CpN patients than in CpP. These results are in keeping with an important role of portal insulin in GH-induced hepatic IGF-I secretion (^19^).

## LOW-LSG STATES II: LIVER FAILURE AND IGF-1 DEFICIENCY

Cirrhosis combines two hits: too few functioning hepatocytes to make IGF-1, and, as glucose and insulin fall, declining portal insulin that renders the surviving hepatocytes even more GH-resistant (^8^,^20^). The result is severe IGF-1 deficiency with GH driven far into the acromegaly range, producing a catabolic state of proteolysis and lipolysis comparable to extended starvation and contributing directly to sarcopenia and progressive malnutrition (^21^,^22^).

The clinical relevance of IGF-1 in this context has become increasingly clear. In advanced chronic liver disease, IGF-1 signaling tracks hepatic dysfunction and fibrogenesis and independently predicts liver-related mortality, supporting IGF-1 as a prognostic biomarker rather than a bystander (^23^). Replacement strategies remain investigational: a pilot randomized trial reported improved albumin with low-dose IGF-1 (^24^), and rodent gene-therapy models show antifibrotic and pro-regenerative effects (^25^). However, large-scale human efficacy data are absent and the safety profile of rhIGF-1 constrains where it could reasonably be used. In severe primary IGF-1 deficiency, the population with the longest exposure, mecasermin causes hypoglycemia in a quarter to two-fifths of treated patients, together with injection-site lipohypertrophy, lymphoid and tonsillar hypertrophy, and rarely intracranial hypertension (^26^,^27^). Two features of cirrhosis amplify the first of these: hepatic glycogen reserve and gluconeogenic capacity are already reduced, so hypoglycemia is common at baseline (^20^), and low IGFBP-3 and acid-labile subunit concentrations mean that any given dose delivers a disproportionately large free IGF-1 fraction (^9^). Efficacy expectations should equally be tempered by evidence of target-tissue resistance: normalizing serum IGF-1 for one week in patients with alcoholic cirrhosis produced no change in functional hepatic nitrogen clearance, although it also produced no nitrogen accumulation and therefore no signal of encephalopathy risk (^28^). The theoretical oncological objection is less straightforward than it first appears, since low rather than high serum IGF-1 predicts the development of hepatocellular carcinoma in hepatitis C-related cirrhosis (^29^). The plausible niche is consequently narrow and should be defined prospectively: Child-Pugh B disease with documented sarcopenia, stable glycemia, and no hepatocellular carcinoma under standard surveillance, with muscle mass, frailty index, and decompensation events rather than albumin as endpoints, and with a bridge-to-transplant indication as the most defensible first target. Until such a trial exists, IGF-1 in cirrhosis is best used as a prognostic stratifier (^23^) rather than as a treatment. Read through the LSG lens, IGF-1 replacement is an attempt to bypass a broken dimmer-and-bulb assembly by supplying the output directly, which is defensible only where the assembly itself cannot be repaired.

## HIGH-LSG STATES: TYPE 2 DIABETES, OBESITY, AND THE WEIGHT-GAIN TRAP

In type 2 diabetes and obesity, portal insulin is high to compensate for insulin resistance, so the liver is GH-hypersensitive: it needs little GH to produce adequate IGF-1, and the GH/insulin ratio falls (^30^,^31^). Low GH status in obesity is largely functional and reverses with sustained weight loss (^32^). The therapeutically important corollary is that any agent which further raises endogenous portal insulin signaling will deepen the low-GH/high-insulin imbalance, and adipocytes facing that ratio can only store, not mobilize, lipid, the ideal physiology for weight gain.

**Figure 3:**
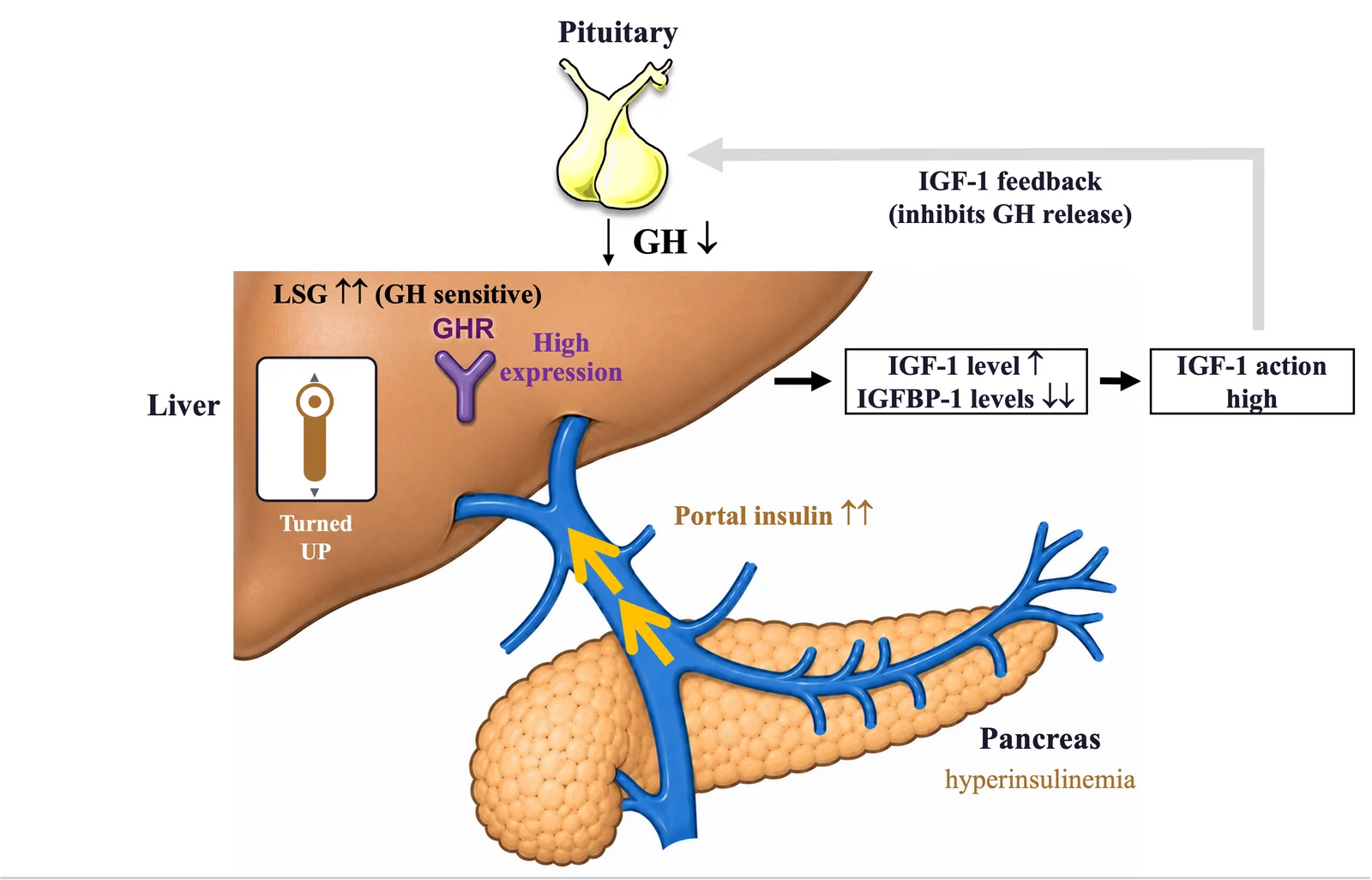
**Early type 2 diabetes mellitus and obesity.** Insulin resistance drives portal hyperinsulinemia, turning the LSG slider up: a GH-hypersensitive liver produces ample IGF-1 from little GH, and strong IGF-1 feedback suppresses GH secretion. The resulting low GH/high insulin ratio disables adipocyte lipolysis and favors fat storage, which is why agents that further raise endogenous portal insulin promote weight gain and insulin-sensitizing agents are preferable. GH: growth hormone; GHR: GH receptor; IGF-1: insulin-like growth factor-1; IGFBP-1: IGF-binding protein-1; LSG: liver sensitivity to GH (portal-insulin-set hepatic GHR availability). Arrow thickness denotes the magnitude of each flux; the vertical LSG slider denotes hepatic GH-receptor availability; the orange arrows are the portal insulin setting the slider; the gray arrow denotes IGF-1 negative feedback inhibiting GH release from the pituitary.

This predicts the differential weight effect of antidiabetic drug classes. Sulfonylureas and PPAR-γ agonists, which raise endogenous insulin or insulin signaling, promote weight gain even without increased caloric intake (^33^), whereas insulin-sensitizing approaches and agents that do not push portal insulin are the rational metabolic choice. The incretin classes complicate and enrich this picture. GLP-1 receptor agonists, now first-line where atherosclerotic cardiovascular disease or heart failure coexists with type 2 diabetes (^34^), produce a discordant GH/IGF-1 signature: chronic treatment increases GH secretion while total IGF-1 falls modestly, plausibly via reduced IGFBP-3 and shifts in hepatic GH sensitivity (^35^,^36^). Mechanistically this is what the LSG framework predicts when a therapy improves insulin sensitivity and reduces the portal insulin demand: the dimmer is turned down, GH secretion rises to compensate, and the resulting GH-IGF-1 discordance reflects altered hepatic responsiveness rather than a biological paradox. The next-generation multi-agonists, including molecules that deliberately engage the IGF-1 receptor alongside incretin receptors, places the GH/IGF-1 axis an intentional therapeutic target rather than an incidental consequence of treatment (^37^) (**Figure 3**).

## THE INTEGRATIVE CASE: ACROMEGALY AS MAXIMAL LSG DYSREGULATION

Acromegaly is where every mechanism in this review converges. The somatotroph adenoma drives GH excess, which induces insulin resistance and systemic and portal hyperinsulinemia. The high portal insulin makes an already GH-flooded liver even more GH-sensitive, thereby amplifying IGF-1 production. In parallel, hyperinsulinemia-driven suppression of IGFBP-1 raises the free, active IGF-1 fraction on top of that (^14^,^38^,^39^). The dimmer is jammed wide open while the generator continues to overproduce current.

The power of the insulin contribution is illustrated by an old observation with a modern echo: hypoinsulinemia induced by prolonged fasting can completely normalize serum IGF-1 in acromegaly (^40^), and a eucaloric very-low-carbohydrate ketogenic diet, which lowers insulin, produces the same directional effect (^41^). GH also has concentration-dependent tissue effects, requiring less GH for gluconeogenesis than for IGF-1 generation. Thus, systemic hyperinsulinemia can blunt peripheral lipolysis while the portal compartment stays under GH control and keeps driving hyperglycemia (^14^,^42^).

**Figure 4:**
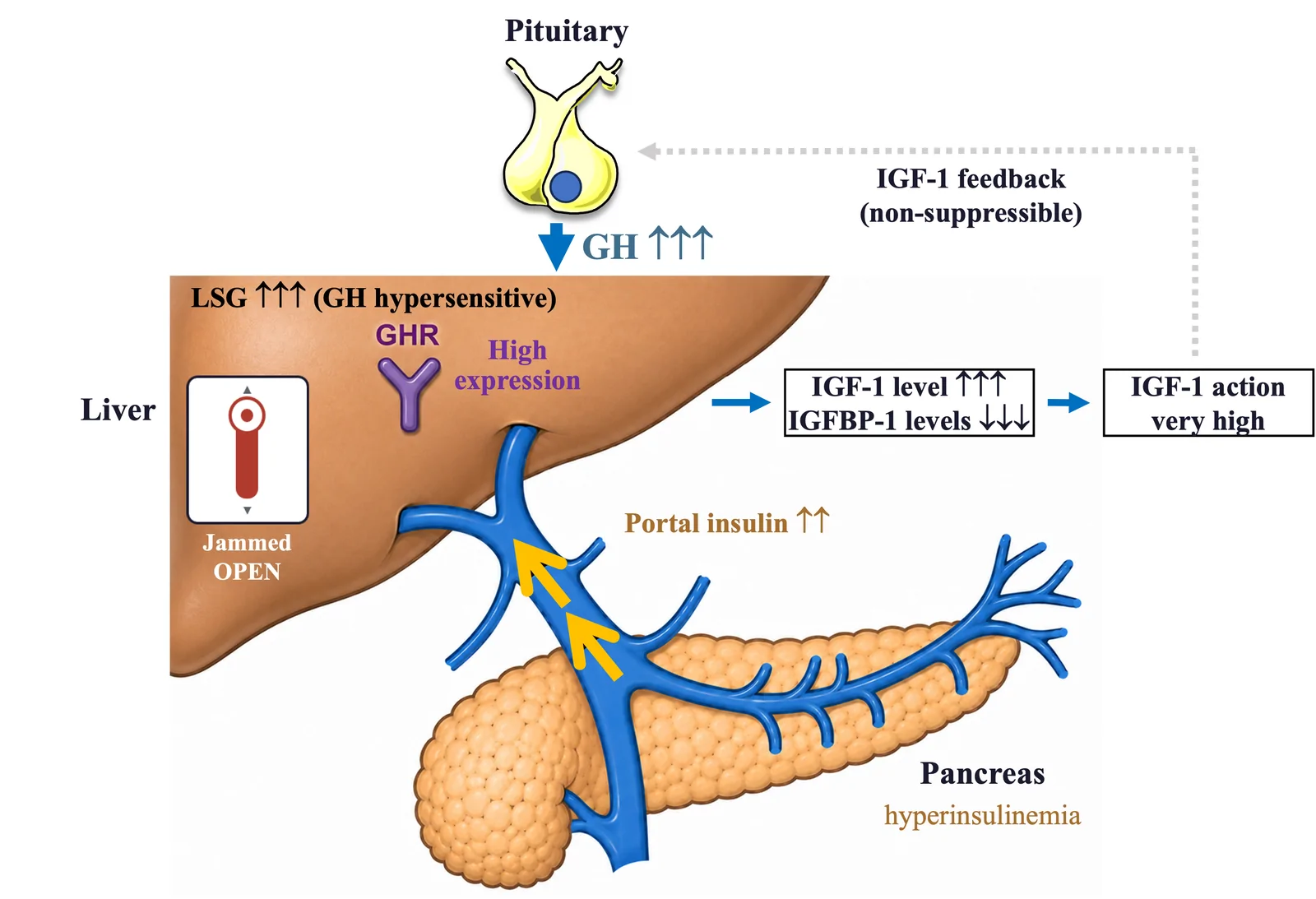
**Acromegaly.** The somatotroph adenoma floods the liver with autonomous GH that is not restrained by feedback. GH-induced insulin resistance raises portal insulin, turning the LSG slider up and rendering the GH-flooded liver even more GH-sensitive; concurrent IGFBP-1 suppression raises the free, bioactive IGF-1 fraction. Every arm points the same way, so IGF-1 normalization alone is an incomplete treatment target. That lowering insulin by prolonged fasting or a very-low-carbohydrate diet can normalize IGF-1 confirms the strength of the insulin arm. GH: growth hormone; GHR: GH receptor; IGF-1: insulin-like growth factor-1; IGFBP-1: IGF-binding protein-1; LSG: liver sensitivity to GH (portal-insulin-set hepatic GHR availability). Arrow thickness denotes the magnitude of each flux; the vertical LSG slider denotes hepatic GH-receptor availability; the orange arrows are the portal insulin setting the slider; the dotted gray arrow denotes nos-suppressible IGF-1 negative feedback on the pituitary.

This is why IGF-1 normalization alone is an inadequate treatment target. Therapies differ sharply in their metabolic footprint even at equivalent IGF-1 control. The GHR antagonist pegvisomant blocks GH action without suppressing insulin secretion and consistently improves insulin sensitivity, fasting glucose, and glycosylated hemoglobin (^43^,^44^). First-generation somatostatin receptor ligands are broadly glucose-neutral, whereas the second-generation ligand pasireotide frequently worsens glycemic control by suppressing insulin secretion, an effect reversible on discontinuation. In contrast, dopamine agonists tend to have more favorable effects (^45^,^46^). A patient brought to identical serum IGF-1 level targets on pasireotide versus pegvisomant can therefore occupy opposite ends of the glucose-metabolism spectrum. Choosing therapy on serum IGF-1 alone, blind to where each drug sets portal insulin and hepatic GH sensitivity, leaves half the clinical picture unmanaged (**Figure 4**).

## ESTIMATING LSG IN CLINICAL PRACTICE

Portal insulin is not measurable outside the research setting, so clinical use of the LSG concept depends on proxies, and three are already available. Fasting IGFBP-1 is the closest single circulating readout of hepatic insulin exposure: serum IGFBP-1 and sex hormone-binding globulin follow estimated portal insulin concentrations rather than whole-body insulin sensitivity, and are appropriate markers of sensitivity only where endogenous insulin secretion is intact (^47^), while fasting IGFBP-1 correlates with clamp-derived hepatic insulin sensitivity more closely than insulin or C-peptide, and does so independently of adiposity (^48^). High IGFBP-1 therefore indicates a low-LSG liver and suppressed IGFBP-1 a high-LSG liver, with the added advantage that IGFBP-1 simultaneously reports the free IGF-1 fraction. Second, C-peptide indexes endogenous and therefore portal insulin delivery, which peripheral insulin cannot do, since the liver extracts up to 85% of insulin at first pass (^12^). The functional confirmation is already contained in this review: the IGF-1 increment after rhGH was significantly smaller in C-peptide-negative than in C-peptide-positive patients (^19^). Third, IGF-1 should be read against a concurrent GH rather than alone. Low IGF-1 with elevated GH identifies the GH-resistant, low-LSG liver; normal or high IGF-1 with suppressed GH identifies the GH-hypersensitive, high-LSG liver of obesity and type 2 diabetes. Where a dynamic measure is preferred, the IGF-1 generation test serves as a functional LSG probe, since hyperinsulinemic obese subjects generate a larger IGF-1 response to exogenous GH than lean subjects (^12^). We propose the combination of fasting IGFBP-1, C-peptide, and the IGF-1/GH pair as a practical bedside estimate of LSG, while emphasizing that none of these proxies has been validated against directly measured portal insulin in humans. Prospective validation against clamp-derived measures of hepatic insulin action is required before such an estimate is allowed to guide treatment.

## THERAPEUTIC IMPLICATIONS AND FUTURE DIRECTIONS

Three practical conclusions follow. First, in acromegaly, treatment selection should weigh the metabolic phenotype alongside IGF-1: pegvisomant or a dopamine agonist for the patient with glucose intolerance and caution should be exerted with pasireotide treatment where diabetes is present (^43^,^45^,^46^). Second, in type 2 diabetes the GH/insulin ratio reframes drug choice: preferred agents are those that lower rather than raise portal insulin demand, while noting the GH/IGF-1 discordance seen with GLP-1 receptor agonists as expected rather than alarming.(^35^-^37^) Third, in cirrhosis, IGF-1 deserves attention both as a prognostic marker and as well as a therapeutic candidate, awaiting adequately powered trials (^23^,^24^).

More broadly, the field would benefit from routinely interpreting the GH/IGF-1 axis in light of portal insulin status rather than treating circulating IGF-1 as a context-free readout of GH activity. The emergence of multi-agonist metabolic drugs that intentionally engage the IGF-1 receptor (^37^), together with the development of GHR-directed and IGF-axis-directed agents in oncology and metabolism, makes a portal-insulin-aware framework increasingly important for predicting both on- and off-target metabolic effects.

## CONCLUSIONS

GH is not simply a hormone that raises circulating IGF-1 levels. Whether GH is permitted to generate hepatic IGF-1 is determined by the insulin that reaches the liver through the portal vein, a variable capture by what we have called liver sensitivity to GH (LSG). Placing type 1 and type 2 diabetes, obesity, liver failure, and acromegaly along a single LSG continuum explains their otherwise disconnected GH/IGF-1/insulin signatures, exposes the portal-systemic compartment error that limits conventional insulin therapy in type 1 diabetes, and reframes treatment selection in both diabetes and acromegaly around metabolic phenotype rather than glycosylated hemoglobin and IGF-1 alone. For the treating physician, reasoning from the dimmer rather than from the bulb is the difference between describing metabolism and understanding its regulation.

## Data Availability

datasets related to this article will be available upon request to the corresponding author.
